# An EBG-Based Triple-Band Wearable Antenna for WBAN Applications

**DOI:** 10.3390/mi13111938

**Published:** 2022-11-10

**Authors:** Rongqiang Li, Chuan Wu, Xiaofeng Sun, Yuan Zhao, Wei Luo

**Affiliations:** 1 College of Electronic Engineering, Chengdu University of Information Technology, Chengdu 610225, China; 2 School of Electronic Science and Engineering, University of Electronic Science and Technology of China, Chengdu 610054, China

**Keywords:** wearable antenna, monopole antenna, EBG, WBAN, SAR

## Abstract

In this article, a triple-band wearable monopole antenna fed by a coplanar waveguide (CPW) with an integrated electromagnetic bandgap (EBG) array is proposed. The monopole antenna consists of an asymmetric inverted U-shaped strip, a horizontal branch, and an L-shaped ground stub, which can generate the 2.45/5.8 GHz wireless local area network (WLAN) band and the 3.5 GHz worldwide interoperability for microwave access (WiMAX) band. To reduce the influence of antenna radiation on the human body, a triple-band 3 × 3 EBG array has been integrated into the back of the monopole antenna. The EBG unit is composed of two rectangular rings and a circular ring, and the operating frequencies correspond to the triple bands of the monopole antenna. In this paper, the impedance and radiation performances of the stand-alone monopole antenna and the integrated antenna are analyzed, and the safety for the human body is evaluated based on specific absorption rate (SAR) values. The proposed triple-band antenna can be used in wearable devices in wireless body area networks (WBANs).

## 1. Introduction

Wireless body area networks have been widely used in recent years, including in health, entertainment, business, military, and other fields [1,2]. As a key element in WBANs, the wearable antenna is usually worn on the human body, e.g., attached to clothing, a helmet, or the wrist [3,4,5]. Human tissues usually have high dielectric constants and nonuniform characteristics, which greatly affect the performances of wearable antennas. Similarly, wearable antennas also have an impact on the human body and so need to meet the established safety standards; they are usually evaluated in terms of SAR values. In recent years, metamaterial (MTM) structures have been proven to effectively reduce the radiation of antennas to the human body and SAR values, including artificial magnetic conductor (AMC) structures [6,7], EBG structures [8,9], and metamaterial surface (MS) structures [10]. References [6,7] describe wearable antennas using single-band AMC structures and references [8,9] wearable antennas loaded with dual-band EBG structures, while reference [10] proposes an antenna with an MS structure. However, these metamaterial structures do not involve multiple operating frequency bands.

With the development of wireless body area networks, there are more and more functional requirements for wearable devices. Antennas operating in multi-band can meet these requirements and have more compact structures than combinations of multiple single-band antennas. Currently, some wearable dual-band antennas have been developed [8,9,11,12]. Reference [11] proposed a dual-band wearable antenna without loading metamaterials. References [8,9,12] proposed a variety of dual-band wearable antennas loaded with metamaterial structures. To further increase the number of bands, some triple-band and multi-band wearable antennas have also been reported [13,14,15,16,17,18]. Reference [13] presented a triple-band antenna with a periodic square groove on the ground and a Hilbert-shaped array for the radiating layer, but the safety for the human body has not been evaluated. In [14], a compact, low-profile button antenna for off-body communication was designed with operating frequency bands of 0.867, 2.38, and 5.85 GHz. In [15], a triple-band open-ring antenna was proposed for ISM, military, and WiMAX wearable applications. In [16], a tri-band dual-polarized multiple-input multiple-output belt-strap antenna was realized for intelligent Internet of Medical Things. In [17], a wearable multi-band CPW-fed slot dipole antenna was incorporated for WBAN applications, which covers the 2.4/5.2/5.8 GHz WLAN, 3.5 GHz WiMAX, and 4.4 GHz C-bands. Reference [18] designed a hepta-band antenna, consisting of a slotted radiator and a 7 × 7 array of periodic square patches. This antenna utilizes the inductive ground plane to reduce the SAR value. It can be seen that most of the current research on wearable antennas with metamaterial structures focuses on the design of single-band or dual-band antennas and rarely involves triple-band or multi-band antennas. Furthermore, triple-band or multi-band metamaterial structures have not been used to improve the performance of existing wearable antennas.

In this paper, a novel triple-band wearable antenna with an integrated triple-band EBG structure is proposed. To the best of our knowledge, this is the first time that a multi-band EBG structure has been used in a multi-band wearable antenna. The impedance bandwidth, radiation performance, and specific absorption rate of the stand-alone monopole antenna and the integrated antenna are analyzed accordingly.

## 2. Antenna Design

### 2.1. Triple-Band Antenna Design

**Figure 1:**
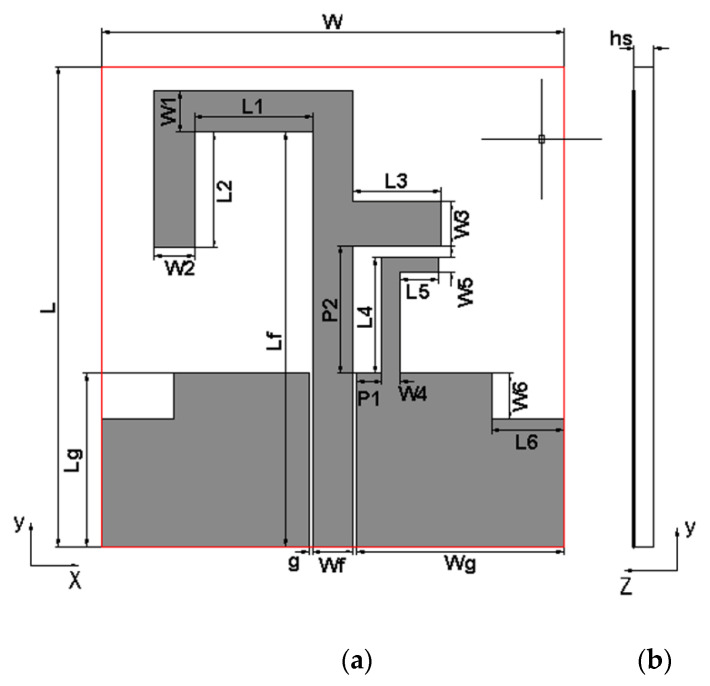
The geometry of the proposed monopole antenna. (**a**) Front view. (**b**) Side view.

**Figure 2:**
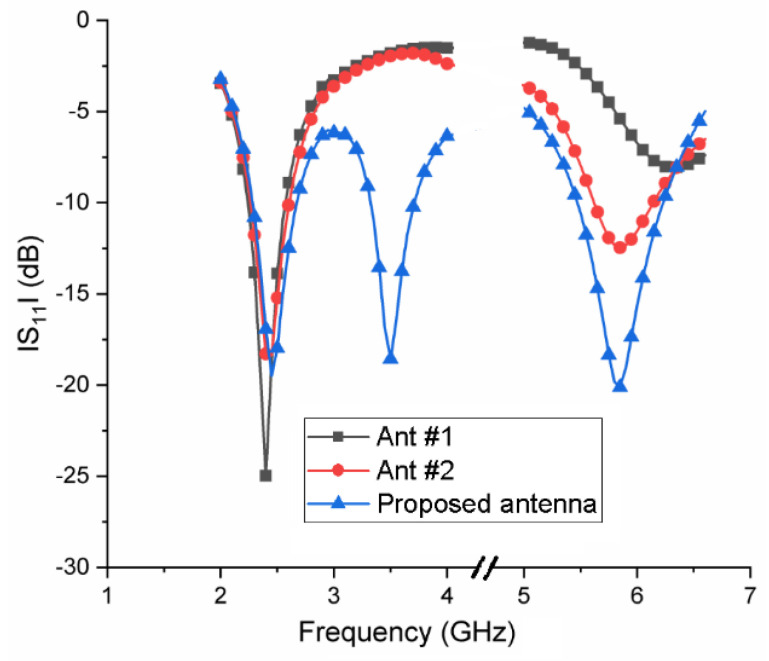
Simulated reflection coefficients of two prototype antennas and the proposed antenna.

The structure of the proposed triple-band monopole antenna is shown in Figure 1. The antenna is fed by a coplanar waveguide (CPW) with a characteristic impedance of 50 Ω, and the ground structure and the radiating surface are located in the same plane. The antenna is composed of an asymmetric inverted U-shaped strip, a horizontal branch, and an L-shaped ground stub, which can form three current paths and generate three operating frequency bands. To further improve the impedance-matching performance of the antenna, two rectangular notches have been cut in the ground plane’s upper-left and upper-right corners. The proposed triple-band antenna was fabricated on a Rogers RO4350 dielectric substrate with a relative dielectric constant of 3.48 and a thickness of 1.5 mm. Optimized by high-frequency electromagnetic simulation software, the overall size of the monopole antenna is 26 × 25 × 1.5 mm^3^, and the dimensions of other parameters are shown in Table 1. The reflection coefficient of the monopole antenna is shown in Figure 2. The −10 dB impedance bandwidth of the antenna is 2.28–2.67 GHz, 3.32–3.71 GHz, and 5.47–6.23 GHz, which can cover the three required frequency bands.

**Figure 3:**
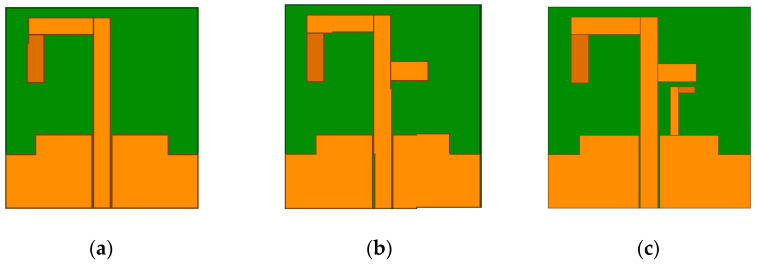
Evolution of the proposed design. (**a**) Ant #1. (**b**) Ant #2. (**c**) Proposed antenna.

To clarify the working principle of the triple-band antenna, Figure 3 shows the design process of the triple-band antenna, and the corresponding reflection coefficients are also given in Figure 2. As can be seen from Figure 2, Ant #1 is an asymmetric inverted U-shaped monopole antenna, which operates in the 2.4 GHz frequency band; Ant #2 is formed by adding a horizontal branch to the radiating strip of Ant #1, resulting in a 5.8 GHz frequency band. Finally, an L-shaped ground stub is added to the CPW ground structure of Ant #2, which will create a 3.5 GHz frequency band to obtain the proposed antenna.

**Figure 4:**
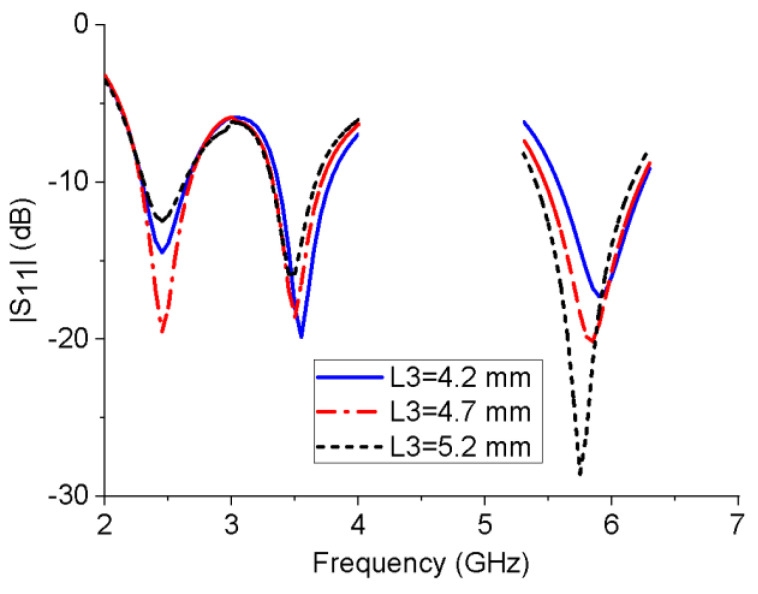
Reflection coefficients of the monopole antenna with different L3 values.

**Figure 5:**
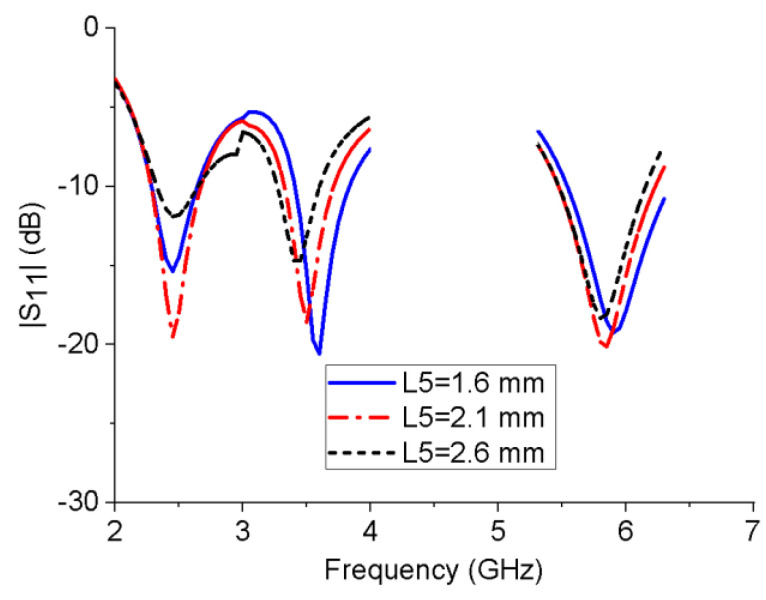
Reflection coefficients of the monopole antenna with different L5 values.

**Figure 6:**
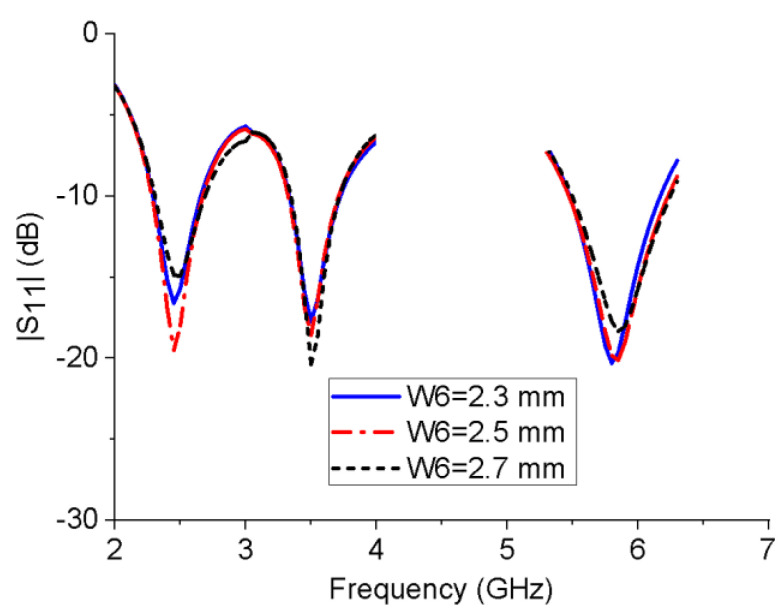
Reflection coefficients of the monopole antenna with different W6 values.

To analyze the influences of several key structural parameters on the impedance bandwidth of the proposed monopole antenna, a parametric study was performed. Figure 4 shows the influence of the horizontal branch length L3 on the reflection coefficient of the antenna. When L3 increases, the resonant frequency of the 5.8 GHz band will shift to a lower frequency, while L3 has much less impact on the 2.45 and 3.5 GHz bands. Similarly, with the increase in the length of the L-shaped ground stub L5, the resonant frequency of the 3.5 GHz band gradually decreases, while the resonant frequency of the other two bands is unchanged, as shown in Figure 5. This is consistent with the working mechanism of the antenna shown in Figure 2 and Figure 3. It can be seen from the Figure 6 that W6 mainly affects the impedance-matching performance of the three frequency bands without changing their resonant frequencies. The further simulation also shows that the rectangular notch length has a similar performance.

### 2.2. Triple-Band EBG Unit

**Figure 7:**
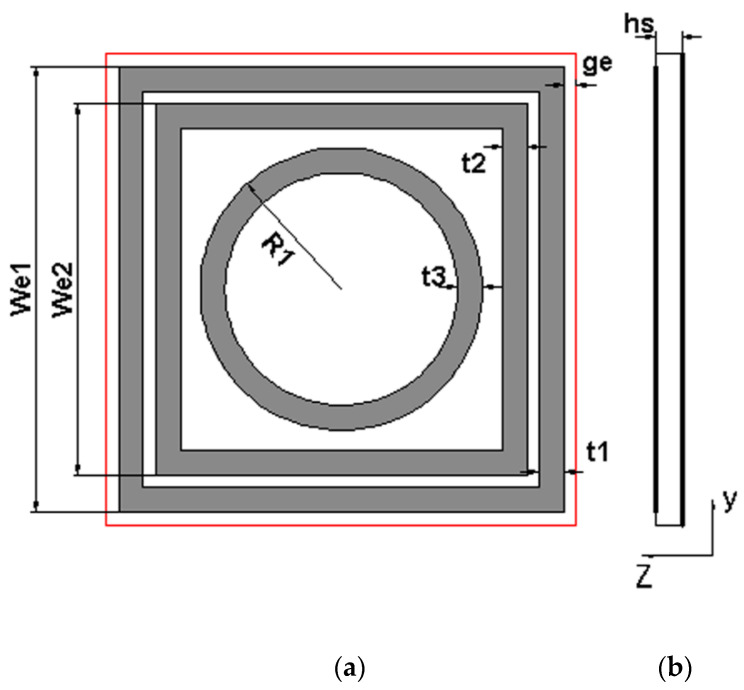
The geometry of the proposed EBG unit. (**a**) Front view. (**b**) Side view.

**Figure 8:**
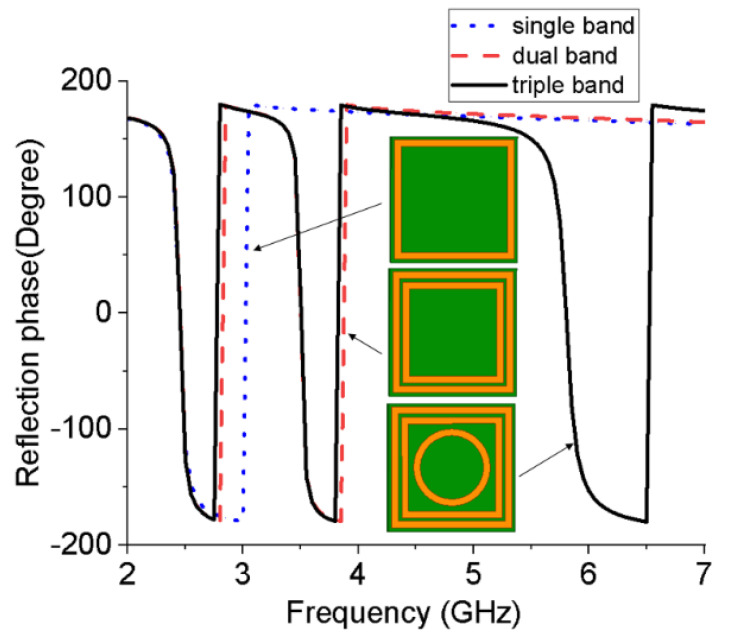
Reflection phase of the proposed triple-band EBG unit compared with the single-band and dual-band EBG.

This paper proposes a new triple-band EBG unit, which uses the same substrate as the antenna. The top metal layer of the substrate is composed of two square rings and a circular ring, and the bottom surface is a fully covered metal layer, as shown in Figure 7. The size of the square metal ring in the EBG unit can be obtained from the equivalent circuit model [19]. Using high-frequency electromagnetic simulation software to design the EBG unit under periodic boundary conditions, its dimensions can be obtained from Table 1. Figure 8 shows the reflective phase of the single-band, dual-band, and triple-band EBG unit structures for comparison, illustrating the design process of the proposed triple-band EBG unit. Usually, multiple EBG units are combined into an array to improve the radiation performance of the wearable antenna.

### 2.3. Integrated Antenna Design

**Figure 9:**
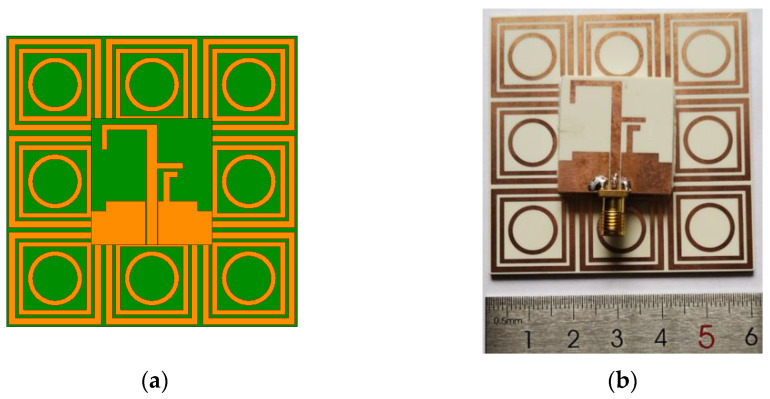
Configuration of the integrated antenna with the EBG array. (**a**) Simulation model. (**b**) Prototype photo.

The proposed triple-band integrated antenna is composed of the monopole antenna (Figure 1) and nine EBG units (Figure 7), as shown in Figure 9; the EBG structure is located below the monopole antenna. To avoid impedance mismatch and short circuits, an air gap of 3 mm is left between the antenna and the EBG structure. Due to the mutual influence between them, the dimensions of the EBG structure and the antenna alone are appropriately adjusted. The overall size of the integrated antenna is 60 × 60 × 6 mm^3^, and the dimensions of the other parameters are shown in Table 2. Figure 9 also shows a photo of the integrated antenna, which was obtained by combining the monopole antenna, the 3 × 3 EBG array, and 3 mm-thick plastic foam. 

## 3. Measurement and Discussion

### 3.1. Reflection Coefficient

**Figure 10:**
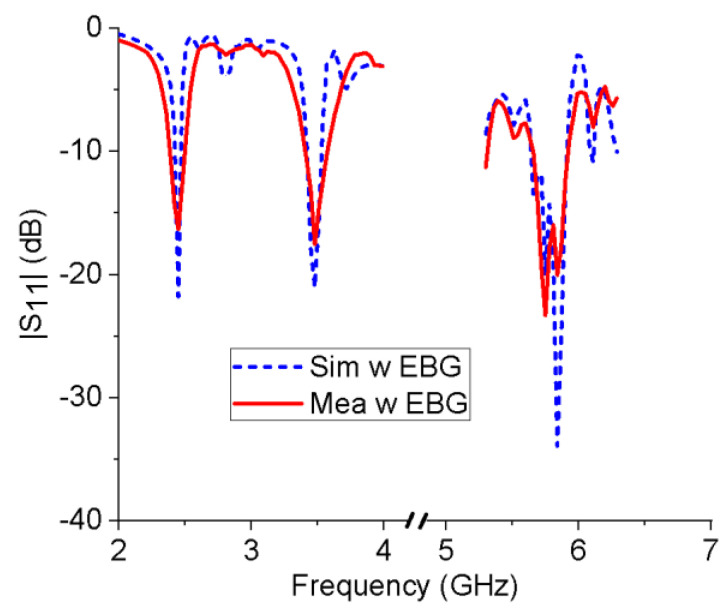
Simulated and measured reflection coefficients of the integrated antenna.

Figure 10 shows the simulated and measured reflection coefficients for the integrated antenna. The measured −10 dB bandwidths are 2.38–2.5 GHz, 3.40–3.59 GHz, and 5.65–5.91 GHz. The measured results are reasonably consistent with the simulation results. However, there are also some discrepancies between them, which may be due to the following factors. First, during the antenna manufacturing process, 3 mm-thick PMI foam was used to replace the air between the antenna and the EBG array and glue them together. Therefore, the plastic foam will introduce some errors. Second, the relative positions of the monopole antenna and the EBG array may not be completely consistent with the simulation settings. Third, the rough soldering of the antenna SMA connector may also cause some errors. 

### 3.2. Radiation Pattern

**Figure 11:**
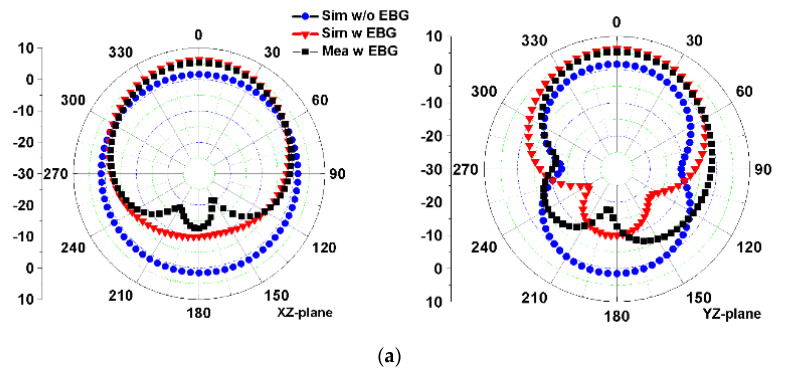

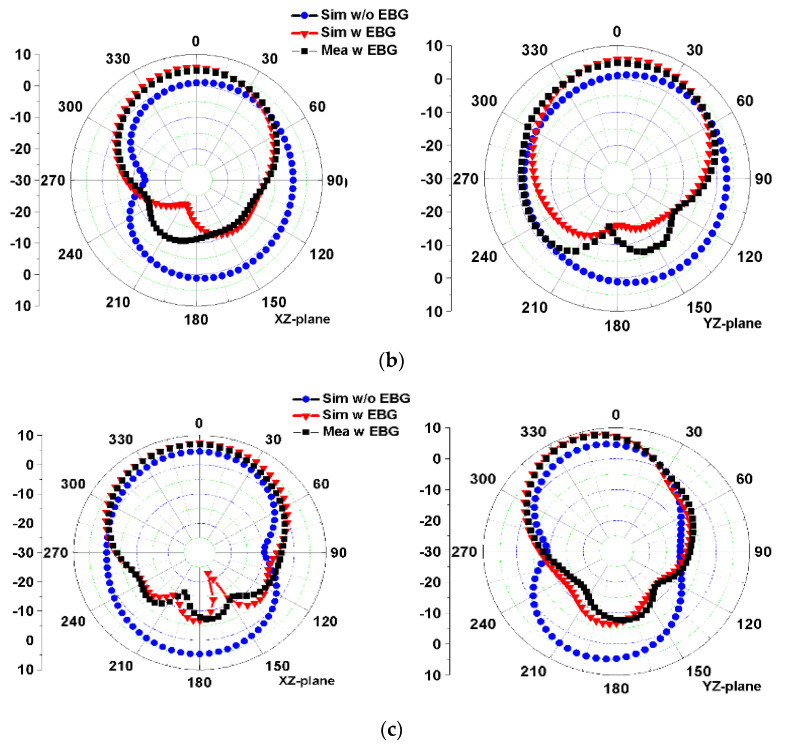
Simulated and measured radiation patterns of the integrated antenna in the xz plane and yz plane at: (**a**) 2.45 GHz, (**b**) 3.5 GHz, and (**c**) 5.8 GHz.

Figure 11 shows the simulated E-plane and H-plane radiation patterns at three resonant frequencies of 2.45 GHz, 3.5 GHz, and 5.8 GHz for the antennas with or without the EBG structure and the measured results of the antenna with the EBG structure. In general, the simulated and measured results agree well. There still are some errors that might have been caused by assembly errors and welding errors. For the monopole antenna without EBG, the radiation patterns on the xz plane (H plane) and yz plane (E plane) at 2.4 GHz and 5.8 GHz are quasi-omnidirectional and 8-shaped, respectively. At 3.5 GHz, the maximum radiation direction of the antenna deviates from the Z axis. According to the simulation, the peak gain of the monopole antenna at 2.45, 3.5, and 5.8 GHz is 1.6, 3.3, and 5.1 dBi, respectively. After the antenna is loaded with the EBG structure, the main radiation direction of the integrated antenna at three resonant frequencies is directed to the Z axis, and the radiations along the –Z axis are significantly reduced, indicating that the EBG structure can effectively improve the front-to-back ratio of the monopole antenna, thereby reducing the radiation of the antenna to the human body and improving the antenna gain. The peak gains of the integrated antenna at 2.45, 3.5, and 5.8 GHz are 6.3, 7.4, and 8.7 dBi, respectively, which are significantly higher than those of the stand-alone monopole antenna without the EBG structure.

### 3.3. SAR Evaluation

**Figure 12:**
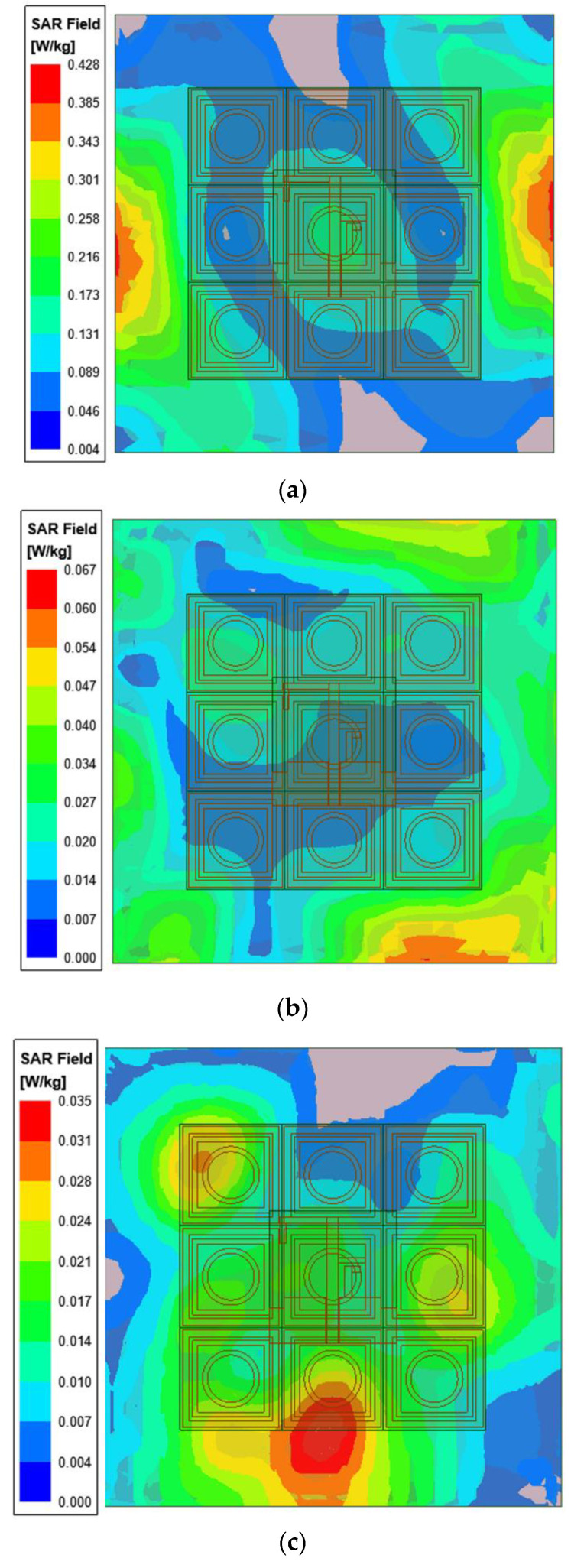
SAR levels of the integrated antenna at: (**a**) 2.45 GHz, (**b**) 3.5 GHz, and (**c**) 5.8 GHz.

Since the wearable antenna is located near the human body, the antenna will radiate to the human body to a certain extent, and the specific absorption rate (SAR) value is usually used to evaluate the safety for the human body. To analyze SAR, a simple three-layer human tissue model was constructed [12]. The model consists of 2 mm-thick skin, 8 mm-thick fat, and 23 mm-thick muscle tissue, with overall dimensions of 90 × 90 × 33 mm^3^. The electrical parameters of the model at different frequencies are consistent with values in the literature [10,12]. The human tissue model was placed on the back of the 3 × 3 EBG array at a distance of 10 mm. The input power to calculate the SAR value was 0.1 W (rms). Figure 12 shows the SAR distribution of the skin tissue at 2.45, 3.5, and 5.8 GHz, and the SAR values were 0.428, 0.067, and 0.035 w/kg, respectively. The simulations showed that fat and muscle tissues have smaller SAR values because they are farther from the antenna. The above SAR values are all less than 1.6 w/kg, which complies with the American standard.

Table 3 provides a performance comparison for the proposed antenna and other reported works. In these works, MTM structures were not used, except for [13]. In [13], the MTM was used as an antenna radiator, but SAR values were not studied. In our work, the EBG structure is used to improve antenna radiation performance and decrease SAR. In general, compared with other reported similar triple-band antennas, the proposed antenna has adequate bandwidth, high gain, and a low SAR.

## 4. Conclusions

In this paper, a triple-band monopole antenna with a 3 × 3 EBG array has been proposed which operates in the 2.45/5.8 GHz WLAN band and the 3.5 GHz WiMAX band. The monopole antenna consists of multiple branches, while the EBG units contain three metal loops. The impedance and radiation performances of the stand-alone monopole antenna and the integrated antenna with a 3 × 3 EBG array were studied and analyzed. The results showed that, compared with the stand-alone antenna, the integrated antenna has a narrower impedance bandwidth, better front-to-back ratio, and higher gain, and its radiation is directed away from the human body. In addition, SAR values for the integrated antenna loaded with a human tissue model were evaluated, and the results showed that the antenna conforms to human safety standards. Compared with other similar reported triple-band antennas, the proposed antenna has adequate bandwidth, high gain, and a low SAR, and it is suitable for wearable devices in wireless body area networks.

## Figures and Tables

**Table 1:** The dimensions of the monopole antenna and EBG unit.

Symbol	Value (mm)	Symbol	Value (mm)	Symbol	Value (mm)
W	25	W3	2.4	Lg	9.4
L	26	L3	4.7	Wg	11.2
Wf	2.2	W4	1.0	g	0.2
Lf	22.5	L4	6.3	We1	18
W1	2.2	P2	6.9	We2	15
L1	6.4	W5	0.8	R1	5.7
P1	1.3	L5	2.1	ge	0.5
W2	2.2	W6	2.5	t1	1
L2	6.3	L6	3.9	t2/t3	1.0

**Table 2:** Design parameters of the integrated antenna.

Symbol	Value (mm)	Symbol	Value (mm)	Symbol	Value (mm)
W	25	W3	1.1	Lg	9.4
L	26	L3	5.4	Wg	11.2
Wf	2.2	W4	1.3	g	0.2
Lf	23.6	L4	6.2	We1	19
W1	1.3	P2	1.1	We2	15
L1	8.1	W5	1.3	R1	5.6
P1	1.1	L5	1.7	ge	0.5
W2	1.1	W6	2.1	t1	1.3
L2	4.0	L6	3.0	t2/t3	1.0

**Table 3:** Comparison with other triple-band wearable antennas.

Ref.	Frequency (GHz)	FBW (%)	MTM	Gain (dBi)	SAR (W/Kg)
[13]	2.45/3.5/5.8	/	Yes	−5/2.5/4.5	/
[14]	0.8/2.3/5.8	4.3/2.1/2.5	No	2.5/3.52/4.8	0.26/0.57/0.93
[15]	2.45/3.0/3.4	4.4/4.0/6.2	No	4.2/6.5/5.0	0.13/0.09/0.09
[16]	2.45/3.0/5.8	13/13.9/10.8	No	2.3/2.7/3.0	0.93/0.89/0.97
Pro.	2.45/3.5/5.8	4.9/5.4/4.5	Yes	6.3/7.4/8.7	0.4/0.06/0.03

## Data Availability

Not applicable.
